# Protective Effects of Panax Notoginseng Saponins on Cardiovascular Diseases: A Comprehensive Overview of Experimental Studies

**DOI:** 10.1155/2014/204840

**Published:** 2014-07-24

**Authors:** Xiaochen Yang, Xingjiang Xiong, Heran Wang, Jie Wang

**Affiliations:** ^1^Department of Cardiology, Guang'anmen Hospital, China Academy of Chinese Medical Sciences, No. 5 Beixiange, Xicheng District, Beijing 100053, China; ^2^Cancer Research Institute, Central South University, Changsha, Hunan 410078, China

## Abstract

Panax notoginseng saponins (PNS) are one of the most important compounds derived from roots of the herb Panax notoginseng which are traditionally used as a hemostatic medicine to control internal and external bleeding in China for thousands of years. To date, at least twenty saponins were identified and some of them including notoginsenoside R1, ginsenoside Rb1, and ginsenoside Rg1 were researched frequently in the area of cardiovascular protection. However, the protective effects of PNS on cardiovascular diseases based on experimental studies and its underlying mechanisms have not been reviewed systematically. This paper reviewed the pharmacology of PNS and its monomers Rb1, Rg1, and R1 in the treatment for cardiovascular diseases.

## 1. Introduction

**Figure 1:**
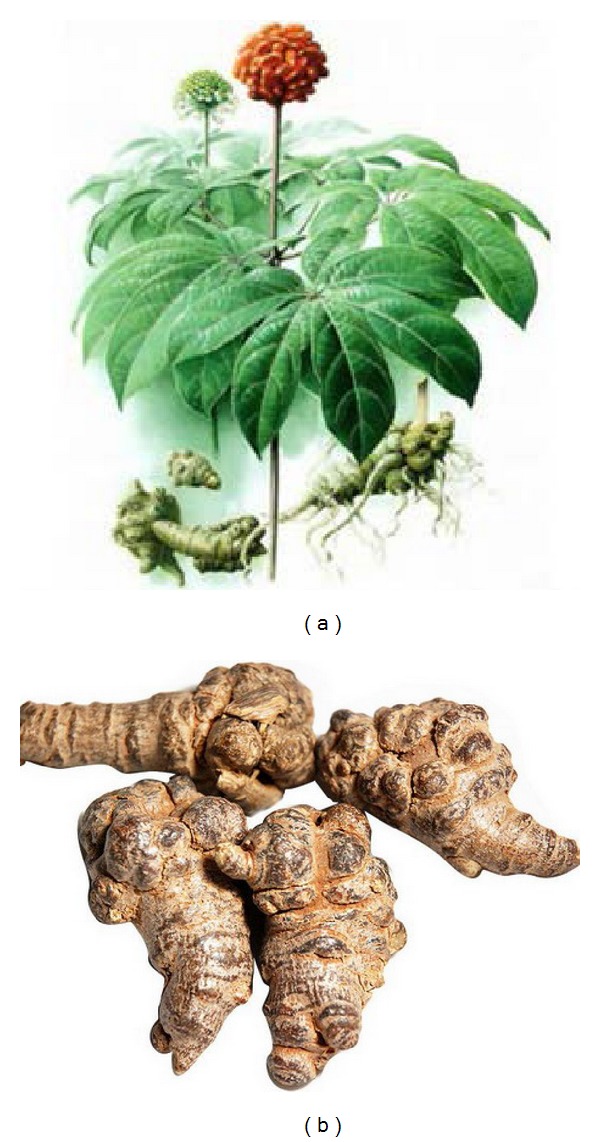
Morphology of Radix notoginseng. (a) Whole plant; (b) roots for pharmaceutical use.

Sanchi, also known as radix notoginseng, is a Chinese herbal medicine (CHM) prepared from roots of the herb Panax notoginseng (see Figure 1). It is traditionally used as a hemostatic medicine to control internal and external bleeding in China for thousands of years. According to the theory of traditional Chinese medicine, Sanchi is sweet, bitter in flavor, and enters the “heart,” “pericardium,” and “liver” channels which function in activating blood circulation of the whole body. With the increasing attention to complementary and alternative medicine, natural products have been clinically used worldwide for the treatment of cardiovascular diseases (CVDs) due to their vasodilatory and antihypertensive actions with good effect. Currently, Sanchi as a commonly used herb for stanch bleeding, invigorating, and supplementing blood has been used for treating CVDs. It is gaining attention increasingly both in developing and in developed countries, including the United States, Japan, and Korea for its efficacy and lower adverse effects. Moreover, it has function of myocardial protection, especially for improving ischemia/reperfusion- (I/R-) induced injury after percutaneous coronary interventional therapy [1].

The chemical constituents of radix notoginseng are complex. As early as the 1930s, some scholars began to study the chemical constituents of radix notoginseng but had slow progress. To the 1970s, with the development of modern test science and technology, this field of research had achieved more and more significant results. Modern researches had showed that radix notoginseng consisted of saponin, dencichine, polysaccharides, amino acids, flavonoids, phytosterols, fatty acids, volatile oils, aliphatic acetylene hydrocarbons, and trace elements [2]. Panax notoginseng saponins (PNS) are one of the main active ingredients of Panax. To date, twenty-seven saponins were identified and nine of them including notoginsenoside R1, ginsenoside Rb1, Rb2, Rb3, Rc, Rd, Re, Rf, and Rg1 were quantified from different parts of Panax [3]. Most of these monomer components are 20(S)-protopanaxadiol and 20(S)-protopanaxatriol. But oleanolic acid-type saponins were not found, which was different from the same plant ginseng and American ginseng [4]. There are also a lot of same monomers compared with ginseng and American ginseng saponins, such as ginsenosides Rb1, Rb2, Rb3, Rc, Rd, Re, and gypenoside. Among them, the content of Rg1 and Rb1 is higher, as the main saponins of radix notoginseng [5]. Ginsenoside Rb1 can promote the formation of nerve fibers and maintain its function to prevent sexual dysfunction, depress central nervous system, promote serum protein synthesis, promote cholesterol synthesis and decomposition, and inhibit the decomposition of neutral fat and antihemolytic reaction [6]. Ginsenoside Rg1 can excite central nervous system, prevent sexual dysfunction, enhance memory, eliminate fatigue, promote DNA and RNA synthesis, and inhibit platelet aggregation [7]. Physiological activity of ginsenoside Rc is mainly inhibiting the central nervous system, so that may cause nerve trance phenomena. Compared with ginseng and American ginseng, Panax consists of more Rb1 and Rg1, but without Rc. Therefore, taking Panax has kind of strong tonic effect, without the ginseng-induced trance-like effect [8].

**Figure 2:**
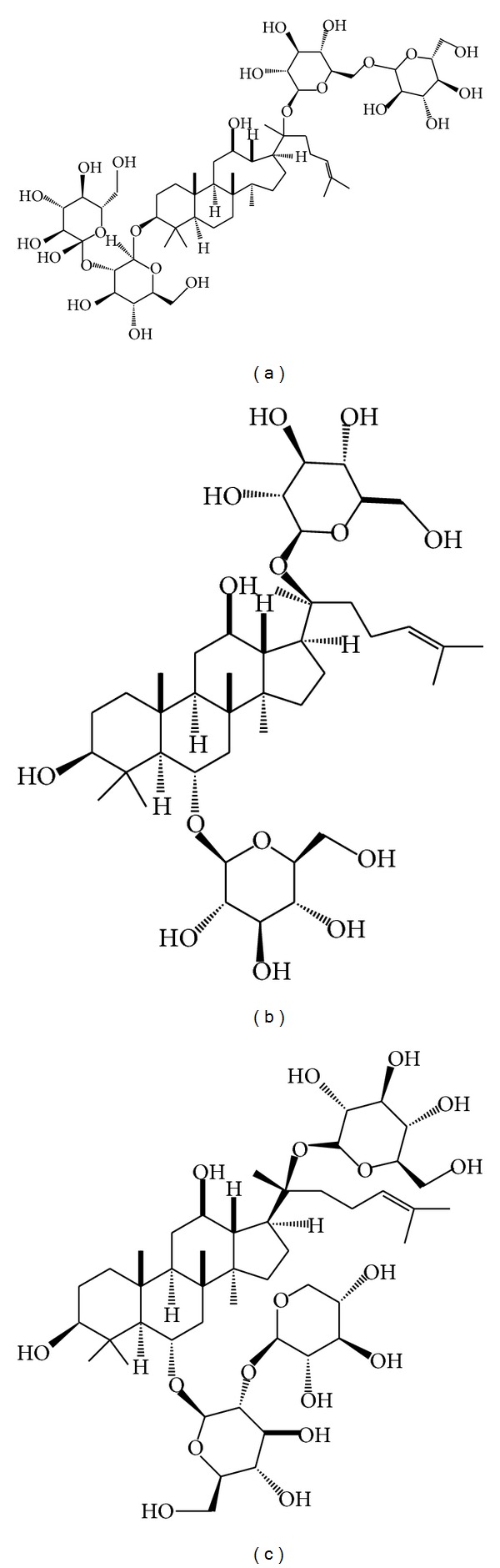
Main compounds of Panax notoginseng saponins. (a) Ginsenoside Rb1; (b) ginsenoside Rg1; (c) ginsenoside R1.

Although the incidence of CVDs is increasing rapidly with the infectious diseases controlled and improvement of people's living, CVDs are still the leading problem for human health [9]. Recently, there is a growing and sustained interest in the benefits of herbal monomer and potential drug interactions with western medications, especially for patients with CVDs for safety and fewer side effects. Protective functions of PNS on the cardiovascular system include inhibition of platelet aggregation, increasing blood flow, improving left ventricular diastolic function in hypertensive patients, and anti-inflammatory effect. These biomarkers are the potential clinical therapeutic targets for cardiovascular disease. The chemical structures of PNS (Rb1, Rg1, and R1) are shown in Figure 2. This paper reviewed the pharmacology of PNS and its monomers Rb1, Rg1, and R1 in the treatment for cardiovascular diseases.

## 2. Cardiovascular Pharmacology

### 2.1. Antiplatelet and Anticoagulant Effect

Long-term antithrombotic therapies, namely, oral antiplatelet agents and anticoagulants, have demonstrated variable clinical effects in cardiovascular diseases, such as coronary heart disease (CHD), hypertension, heart failure, atrial fibrillation, and valvulopathy. Aspirin has been shown to reduce the risk for thrombosis and ischaemic events. However, the possibility of aspirin resistance, which has been described as a number of phenomena, including antithrombotic complications, prolongation of the bleeding time, and inhibition of thromboxane biosynthesis [10], provides an impetus for researching new antiplatelet products with high effectiveness and fewer adverse effects. Sanchi is considered a good source of lead compounds for novel antiplatelet and anticoagulant therapeutics. Notoginsengnosides (NG) isolated from Sanchi could inhibit both the platelet aggregation of platelet rich plasma (PRP) and washed platelet after ADP induction. In the NG-treated platelets, the levels of growth factor receptor-bound protein 2 (Grb2), thrombospondin 1, and tubulin alpha 6 were increased, whereas the levels of thioredoxin, Cu-Zn superoxide dismutase, DJ-1 protein, peroxiredoxin 3, thioredoxin-like protein 2, ribonuclease inhibitor, potassium channel subfamily V member 2, myosin regulatory light chain 9, and laminin receptor 1 were decreased [11]. The analysis of the reactive oxygen species (ROS) level also indicated that NG could decrease the ROS level in platelets. Both raw and steamed Panax notoginseng can significantly inhibit platelet aggregation and plasma coagulation. In addition, steamed Panax notoginseng has significantly more potent antiplatelet and anticoagulant effects than the raw extract, and the antiplatelet and anticoagulant effects increase with increasing steaming durations. Another research [12] demonstrated that the antiplatelet and anticoagulant effects in vitro are positively translated into a prolongation of in vivo rat bleeding time after oral administration of the raw and steamed extracts. Other studies also reported that notoginsenoside R1 (NG-R1) increases the synthesis of tissue-type plasminogen activator (t-PA) and decreases plasminogen activator inhibitor-1 (PAI-1) activity in cultured human endothelial cells from different vascular sources. NG-R1 could increase the fibrinolytic potential in vitro by increasing the production of t-PA and u-PA [13]. This potential effect of NG-R1 on the improvement of fibrinolytic system might contribute to the effect of the Chinese herb drug Panax notoginseng in the treatment of cardiovascular diseases.

### 2.2. Protecting Myocardium Cells from Apoptosis

Increased cardiomyocyte apoptosis can be commonly observed in heart failure after acute myocardial ischemia (MI) [14, 15]. Oxidative stress plays a significant role in tissue necrosis and reperfusion injury during this process [16]. Protecting myocardium cells from apoptosis is a potentially promising approach to limit infarct size during the revascularization of patients with acute MI. The cardioprotective mechanism involves the reduction of oxygen-derived free radicals, prolonged acidosis during early reperfusion, and signal transductions that attenuate the multiple manifestations of reperfusion injury by inhibiting the opening of the mitochondrial permeability transition pore (mPTP), a nonspecific channel localized in the mitochondrial inner membrane [17–20]. Among the distal signal transduction pathways involved in cardioprotection, the activation of reperfusion injury salvage kinases (RISK), such as phosphatidylinositol 3-kinase- (PI3K-) Akt and ERK1/2, has been implicated in the mechanistic link between protection of cardiomyocyte apoptosis and cardioprotection [21, 22]. The protective effect and potential molecular mechanisms of PNS on apoptosis in H9c2 cells in vitro and rat myocardial ischemia injury model in vivo were investigated [23]. However, the effect was blocked in vitro by LY294002, a specific PI3K inhibitor. The antiapoptotic effect of PNS was mediated by stabilizing Deltapsim in H9c2 cells. Furthermore the indices of the left ventricular ejection fractions (EF), left ventricular fractional shortening (FS), left ventricular dimensions at end diastole (LVDd), and left ventricular dimensions at end systole (LVDs) suggested that PNS improved rats' cardiac function. They found that PNS could protect myocardial cells from apoptosis induced by ischemia in both the in vitro and the in vivo models through activating PI3K/Akt signaling pathway. In vitro study [23] also suggested that PNS has a significant effect on Ang II-induced rat cardiomyocytes apoptosis by alleviating intracellular calcium overload. It was found that incubating with Ang II (10(−7) mol × L(−1)) for 48 h increased cardiomyocyte apoptosis; PNS (25, 100 mg × mL(−1)) increased myocyte viability. PNS (50 mg × mL(−1)) significantly decreased this Ang II-induced rat cardiomyocyte apoptosis and decreased fluorescent intensity of intracellular calcium. In vitro experiments [24] assessed the protective effect of PNS against doxorubicin on viability of embryonic rat heart cell H9C2. The results showed that pretreatment with PNS significantly lowered the levels of serum LDH, CK, and CK-MB and normalized myocardial superoxide dismutase, glutathione peroxidase, and catalase activities. Another researcher [25] studied the effects of total PNS and its monomers Rb1 and Rg1 on total ATPase and Na(+)-K(+)-exchanging ATPase of guinea pig heart were studied. The results showed that PNS inhibited the total myocardial ATPase but had no significant effect on the myocardial Na(+)-K(+)-exchanging ATPase.

### 2.3. Promoting Cardiac Angiogenesis

Despite extensive atherosclerosis that precludes complete revascularization, an increasing number of patients are being referred for coronary artery bypass surgery [26]. A variety of novel angiogenic therapies [27–30] are being evaluated as adjuncts to bypass grafting or as sole therapies for patients who are not candidates for coronary artery bypass. Vascular endothelial growth factor (VEGF) and basic fibroblast growth factor have been given as protein therapy or as gene therapy by transfection by naked DNA or with adenoviral vectors [31, 32]. However, the limitations of growth factor therapy include the risks of systemic effects inducing problematic angiogenesis in the retina or the potentiation of growth and metastasis of occult tumors [33]. It was investigated that Radix Notoginseng formula had effects on secretion of vascular endothelial growth factor (VEGF) and expression of vascular endothelial growth factor receptor-2 (VEGFR-2) in human umbilical vein endothelial cells (HUVECs) in vitro [34]. The results showed that Radix Notoginseng formula can promote HUVEC proliferation and secretion of VEGF, as well as the expression of VEGFR-2 protein, which may be one of the mechanisms of Radix Ginseng and Radix Notoginseng formula in promoting angiogenesis with fewer adverse effects.

### 2.4. Antimyocardial Ischemia and Hypoxia Effect

Antimyocardial ischemia/hypoxia are still challenging for treating coronary heart diseases. It has been generally accepted that efficient therapeutic approaches to ischemic heart diseases are to improve the myocardial oxygen balance between supply and demand in the ischemic heart, by either increasing coronary blood flow or decreasing cardiac mechanical function or both [35]. The traditional antianginal drugs included nitrates, b-blockers, and calcium channel blockers, which are thought to improve the myocardial oxygen balance with changes in hemodynamic parameters. However, these drugs also bring further injury to the weak cardiac function [36]. Therefore researchers are trying to find more efficient means with fewer side effects to prevent and treat myocardial ischemia. To investigate the effects of PNS extracts on antimyocardial ischemia injuries in vivo, it was found that PNS may be therapeutically useful for ameliorating antimyocardial ischemia injuries by decreasing oxidative stress and repressing inflammatory cascade [37]. PNS restrained the oxidative stress related to myocardial ischemia injury as evidenced by decreased malondialdehyde (MDA) and elevated superoxide dismutase (SOD) activity. Meanwhile, the inflammatory cascade was inhibited as evidenced by decreased cytokines such as tumor necrosis factor-*α* (TNF-*α*), C-reactive protein (CRP) and interleukin-1*β* (IL-1*β*). Other researches [38] also studied the protective effect and mechanism of ginsenoside Rg1 in cardiomyocytes hypoxia/reoxygenation (H/R) model. The results showed that pretreatment with ginsenoside Rg1 reduced lactate dehydrogenase release and increased cell viability in a dose-dependent manner. Fluorescence analysis also demonstrated ginsenoside Rg1 reduced intracellular ROS and suppressed the intracellular [Ca(2+)] level, which suggested that the myocardial protection of ginsenoside Rg1 during H/R is partially due to its antioxidative effect and intracellular calcium homeostasis.

### 2.5. Lipid-Lowering Effect

Lipid-lowering medications have been shown to reduce both atherogenic lipoproteins and cardiovascular morbidity and mortality [39–43]. Although the efficacy of lipid-lowering medications (various statins) in reducing atherogenic lipoproteins and vascular inflammation varies significantly, the impact of these differences on clinical outcome is unknown [44]. Alternative strategies and target levels for lipid reduction are still an important issue for lowering the risk of cardiovascular events. To explore potential benefits in cardiovascular disorders associated with excess cholesterol and hyperlipidemia, researchers investigated the effects of Sanchi on hyperlipidemia and oxidative stress in male Sprague-Dawley rats maintained on a high-fat die, which results in a significant decline in serum levels of total cholesterol (TC), triglycerides, and low-density lipoprotein-cholesterol, with an increase in serum high-density lipoprotein-cholesterol levels [45]. In addition, the results also showed reduced levels of hepatic HMG-CoA reductase.

### 2.6. Inhibitory Effect on the Inflammatory Responses

Inflammatory response plays an important role in cardiovascular disease including the process of atherosclerosis, cardiac hypertrophy, endothelium injury, and myocardial infarction. Dendritic cells (DCs) play a central role in the regulation of both inflammation and adaptive immunity. Notoginseng extracts have potential ability to modulate toll-like receptor (TLR) ligand-induced activation of cultured DC2.4 cells [46]. The inhibition of TNF-alpha production was time-dependent in LPS-stimulated cells by pretreatment or concurrent treatment of notoginseng but not after delayed addition of the herbal extract. Additionally, ginsenoside Rg1 more effectively inhibited LPS-stimulated cytokine production by DC2.4 cells than ginsenoside Rb1.

### 2.7. Inhibition of Intimal Hyperplasia and Smooth Muscle Cell Proliferation

Abnormal proliferation of vascular smooth muscle cells (VSMCs) plays an important role in formation of atherosclerosis and restenosis. However, mechanisms underlying this effect have not been completely elucidated. Several lines of evidences demonstrated that PNS could inhibit the vascular intimal hyperplasia and the smooth muscle cell (SMC) proliferation, suppress the expression of various growth factors, and induce the differentiation, maturity, and apoptosis of the vascular smooth muscle cell (VSMC). Experiments [47, 48] indicated that PNS could inhibit vessel restenosis after vascular intimal injury, and its mechanisms may be related to the blockage of the excessive proliferation of VSMC. One research [47] showed that PNS significantly inhibited the vascular intimal hyperplasia and significantly lowered the expression of proliferating cell nuclear antigen (PCNA), cyclin E, cyclinD1, fibronect (FN), and matrix metalloproteinase-9 (MMP-9) but had no significant impacts on the expression of collagen I (Col-I) and TIMP-1. Other researches [48] showed that the intimal area (IA), intimal thickness (IT), hyperplasia ratio of intimal area (HRIA), the ratio of intimal/mesolamella area, and thickness were significantly lower after treatment with PNS in rats; meanwhile the expression of proliferating cell nuclear antigen (PCNA) was significantly lowered. Another research [49] showed the restraining impact of PNS on the proliferation of VSMCs and revealed the associated mechanisms through cell cycle-related factors and extracellular regulated protein kinase (ERK) signal transduction pathway. In addition, there were also no significant differences between atorvastatin and PNS on inhibiting the activation of PDGF-induced P-ERK1/2 and increasing the content of MKP-1. PNS both inhibits VSMCs proliferation and induces VSMCs apoptosis through upregulating p53, Bax, and caspase-3 expressions and downregulating Bcl-2 expression, which constitute the pharmacological basis of its antiatherosclerotic action [50]. PNS (100 and 400 micrograms *·* mL^(−1)^) can inhibit the proliferation of VSMC stimulated by hypercholesterolemic serum (HCS) [51]. Another research [52] concluded that PNS can significantly inhibit the VSMC proliferation induced by hyperlipidemia serum. Hyperlipidemia serum could promote VSMC proliferation, while PNS could weaken this effect significantly.

### 2.8. Antiatherosclerosis Effect

Atherosclerosis (AS) is a systemic cardiovascular disease with complicated pathogenesis involving vascular smooth muscle cell (VSMC) proliferation, endothelial dysfunction, lipid deposition, oxidative stress, and chronic inflammation. Endothelial dysfunction plays an important role in the formation of atherosclerosis. The unbalance between the induction of VSMC apoptosis and inhibition of VSMC apoptosis attributes the injury of endothelium. One of the most important substances for inducing VSMC apoptosis is NO, which has vasodilative effect on blood vessels. Various studies had demonstrated that PNS could attenuate atherosclerosis with different mechanisms. To investigate the effects of PNS on antioxidant effect on the formation of atherosclerosis, one research [53] showed that PNS can lower the serum levels of lipid and oxidized low-density lipoprotein (oxLDL), ratio of plaque area to vessel area, and expression of CD40 and MMP-9 in the apolipoprotein E-knockout (apoE-KO) mice. Another in vivo study showed that PNS had an effect in reversing the injuries of human umbilical vascular endothelial cells (HUVEC) induced by oxidized low-density lipoprotein (ox-LDL), improving its activity, elevating the adhesion rate with monocytes, and increasing the protein expression of intercellular adhesion molecule-1 (ICAM-1) in HUVEC [54]. It suggested that the protective effect of PNS for treating arteriosclerosis obliterans (ASO) is probably by way of downregulating the expression of ICAM-1 in endothelial cells and inhibiting the adherence of monocytes to endothelial cells. PNS attenuates atherogenesis through an anti-inflammatory action of decreasing the mRNA expression levels of monocyte chemoattractant protein-1 and nuclear factor-kappaB/p65 in the aorta wall after 8 weeks of treatment in rabbits, and regulation of the blood lipid profile includes decreasing the serum levels of TC, triglyceride, low-density lipoprotein-cholesterol, interleukin-6, and C-reactive protein as well as increasing high-density lipoprotein-cholesterol level significantly [55]. PNS enhanced transcriptional activation of the LXRalpha gene promoter, subsequently upregulated ATP-binding cassette A1 and G1 (ABCA1, ABCG1), and inhibited NF-kappaB DNA binding activity in rat aortas [56]. Accumulative studies had been established to investigate the anti-inflammatory effect on atherosclerosis. The molecular mechanisms responsible for the antiatherosclerotic effects of PNS and the inflammatory response were explored [57]. The results suggested that PNS exerts its therapeutic effects on atherosclerosis through an anti-inflammatory action, including reduction of the gene expression of some inflammatory factors, such as integrins, interleukin- (IL-)18, IL-1beta and matrix metalloproteinases-2 (MMP-2), and MMP-9. In addition, PNS was found to increase the expression of IkappaBalpha, whereas attenuating the expression of NF-kappaB/p65, suggesting that the possible mechanism responsible for the effect of PNS was associated with NF-kappaB signalling pathway, which was accompanied with inflammatory response. PNS inhibits zymosan A induced atherogenesis by suppressing phosphorylation of FAK on threonine 397, integrins expression, and NF-kappaB translocation in rats [58]. The similar trend of the inhibitory effects of PNS and its two major individual ingredients, ginsenoside Rg1 and ginsenoside Rb1, on the TNF-alpha-induced NF-kappaB activation was investigated in human coronary artery endothelial cells (HCAECs) [59]. PNS can promote angiogenesis by regulating the VEGF-KDR/Flk-1 and PI3K-Akt-eNOS signaling pathways in human umbilical vein endothelial cells (HUVECs) [60]. In addition, PNS was also shown to promote changes in the subintestinal vessels in zebrafish as a feature of antiangiogenesis effect.

### 2.9. Antiarrhythmia Effect

Atrial arrhythmia (AA) is the most common complication after coronary artery bypass grafting (CABG). In addition to potentially increased risk of stroke and death, patients with recurrent or persistent AA require additional medications, including systemic anticoagulation [61, 62]. Several pharmacologic agents have been used to prevent AA. Rg1 isolated from saponins of Panax notoginseng had effects on cardiac electrophysiological properties and ventricular fibrillation threshold (VFT) in open-chest dogs [63]. The result showed that Rg1 prolonged sinus node recovery time (SNRT) by 19.1%, AV conduction Wenckebach cycle length (AVWCL) by 7.1%, and ventricular effective refractory period (VERP) by 7.9%, while increased VFT by 19.2%. The cardiac electrophysiological effects of Rg1 were similar to those of amiodarone.

### 2.10. Vasodilative Effect

Hypertension, as an independent predisposing factor for heart failure, coronary artery disease, stroke, renal disease, and peripheral arterial disease, is associated with serious morbidity and mortality [64]. Evaluation of possible resistant hypertension begins with an assessment of adherence to medications. To investigate the inhibition of endothelium-dependent abnormalityin vascular relaxation induced by PNS and its effect on receptor-operated Ca2+ channels in vascular smooth muscle, at least three experiments have been established. One research [65] suggested that PNS could increase Ca2+ level in endothelial cells via the receptor-operated Ca2+ channels in the presence of acetylcholine (ACh) or the nonselective cation channels opened by cyclopiazonic acid (CPA). Another research [66] indicated that ginsenoside-Rd could remarkably inhibit Ca2+ entry through receptor-operated calcium channel (ROCC) and store-operated calcium channel (SOCC) without effects on voltage-dependent inward Ca2+ current (VDCC) and Ca2+ release in vascular smooth muscle cells. Ginsenoside-Rd inhibited cell proliferation and reversed basilar artery remodeling [67], while Rb1 and Rg1 increased endothelial-dependent vessel dilatation through the activation of NO by modulating the PI3K/Akt/eNOS pathway and l-arginine transport in endothelial cell [68].

### 2.11. Inhibition of Left Ventricular Remodeling

Left ventricular remodeling can be caused by acute myocardial infarction and then lead to heart failure. Increase in left ventricular mass (LVM) might be an important pathological process for cardiovascular diseases including hypertension and heart failure. Several factors which are associated with increased LVM have been identified, which include blood pressure, physical activity, and blood viscosity [69, 70]. To investigate the effects of PNS on the overall model and in vitro model of cardiac hypertrophy, it was found that PNS inhibited the markers of cardiac hypertrophy including heart weight/body weight (HW/BW), left ventricular weight/body weight (LVW/BW), and the myofibril diameters (MD) in rats in a dose-dependent manner, but SBP of rats was not obviously influenced [71]. PNS significantly inhibited the norepinephrine (NE) induced increase of surface area and protein content in the cultured myocardial cells, indicating that PNS can prevent the overall model and in vitro model of cardiac hypertrophy in rats associated with its inhibitory action on neurohormonal factor NE, but not on pressure overload. A comparative study [72] was conducted on the action of PNS and captopril in treating SHR. The results demonstrate that PNS helps increase the effect of calcium pump on the membrane of sarcoplasmic reticulum, decrease the myocardial intracellular Ca2+, and reduce the mass of the left ventricular muscle. To research the effects of Panax notoginseng saponins (PNS) on angiotensin-converting enzymes 2 (ACE2) and tumor necrosis factor-alpha (TNF-alpha) in rats with postmyocardial infarction ventricular remodeling, it was concluded that PNS can stimulate ACE2 to inhibit the expression of TNF-alpha and enhance the antioxidants. In addition, PNS can reduce pathological injury of cardiac myocytes in myocardial ischemia and cardiac muscle, which can improve ventricular remodeling [73].

## 3. Conclusion

Cardiovascular disease (CVD) is a major cause of early morbidity and mortality in most developed countries, which is caused by atherosclerosis mainly, and is always treated by western routine drugs for reducing the risk of heart attack and emergency medical care [74]. During the most recent decade, the rate of death due to CVD has declined, but the burden of disease remains high. Although improved medical care and acute management of myocardial infarction have led to a considerable reduction of early mortality rate survivors, an increased prevalence of secondary diseases such as chronic heart failure caused by ventricular remodeling decreased the benefit of medical care [75, 76]. An enormous cost factor for the healthcare system that cardiovascular operations and interventional procedures increased by 28% from 2000 to 2010 was implicated [77]. Therefore, the main issue of current pharmacological, interventional, or operative therapies is their disability to reduce the risk factors and endpoints of cardiovascular diseases. Recently, there is a growing and sustained interest in the benefits of complementary herbal medicine (CHM) and potential drug interactions with western medications, especially for patients with CVD for safety and fewer adverse effects. In China, the research field of integration of traditional and western medicine in treating CVD is developing rapidly for over 30 years. Among them, issue on the blood stasis syndrome (BBS) and promoting blood circulation and removing blood stasis (PBCRBS) is one of the most developed fields of integration of traditional and western medicine. The research development of herbal extracts, herbal single monomer, and traditional Chinese patent medicine (TCPM) is increasing rapidly, especially in integration with routine western medical interventions [78–82]. For instance, at least one TCPM may regularly be used in patients with angina pectoris after percutaneous coronary intervention (PCI) in either western medicine hospitals or traditional Chinese medicine hospitals, because it shows the ability to ameliorate in-stent restenosis after PCI [83].

Several lines of evidence have proved that PNS appears to be a promising natural cardioprotective agent. PNS, which is a member of the major lipophilic components extracted from Panax notoginseng, has indicated significant therapeutic effects and multiple pharmacological actions including antiplatelet, anticoagulant, antithrombotic, antiatherosclerosis, lipid-lowering, vasodilative, anti-inflammation, antiischemia, antiarrhythmia, antihyperplasia, and promoting angiogenesis effects. The identified effect of PNS and its major monomers is summarized in Tables 1 and 2. All that demonstrated that PNS can regulate whole body by acting on multicenter and multitargets for protection. However, there are still some problems existing in the research of PNS for cardiovascular diseases. (1) In the most experimental researches, they focused on the mechanisms of one aspect of PNS, the experimental design owes rigor, and only a few studies were equipped in vitro and in vivo at the same design. (2) Research on antiplatelet and anticoagulant effect and antiatherosclerosis effect was adequate and performed more frequently compared with other effects of studies. However, studies about antiarrhythmic effect and lipid-lowering effect are seldom in vivo or in vitro. For vasodilative effect, there is deficiency of in vivo research to investigate blood pressure lowering induced by PNS. As hypertension is an independent predisposing factor for heart failure, coronary artery disease, stroke, renal disease, and peripheral arterial disease, evaluation of possible resistant hypertension begins with an assessment of adherence to medications. (3) Despite the fact that there were randomized controlled trials (RCTs) about PNS, for instance, Xuesaitong soft capsule and Xuesaitong injection for treating coronary heart diseases, but the methodological and qualities of these kinds of RCTs were not vigorous, it is imperative to conduct multicentered, large-sized samples and randomized and arid controlled trials to reasonably evaluate the efficacy and safety of PNS for CVDs. In addition, there are also short of systematic reviews (SRs) about PNS.

In future research for PNS or another CHM for CVDs, it is necessary to take a systematic study on the mechanism of Chinese herb and formulas for PBCRBS. We could suggest that PNS is an important herbal medicine for cardiovascular protection; nevertheless, it could also be used as an antihypertensive agent on the basis of our clinical experience. It has been proved that PNS has an effect on reversing ventricular hypertrophy, protecting target organs, improving blood vessel function, and other auxiliary vasodilator effects. Therefore, further in vivo researches are needed to explore and verify the potential effect to provide precise guidance for clinical use and new drug discovery. Meanwhile, more rigorous studies could be performed to prove the efficacy of PNS for treating cardiovascular diseases by translating these pharmaceutical effects into clinical practice, in order to indicate its multitarget actions and finally improve clinical efficacy.

## Figures and Tables

**Table 1:** Cardiovascular effects of PNS (Rb1, Rg1, and R1) in vitro research.

Compounds	Cells/tissues	Effects		Study
NG	PRP and rat washed platelets	Antiplatelet, anticoagulant, and antithrombotic effect	(i) Inhibit ADP-induced platelet aggregation	Yao et al., 2008 [3]
NG	Rat washed platelets		(ii) Increase the levels of Grb2, thrombospondin 1, and tubulin alpha 6 and decrease the levels of thioredoxin, Cu-Zn superoxide dismutase, DJ-1 protein, peroxiredoxin 3, thioredoxin-like protein 2, ribonuclease inhibitor, potassium channel subfamily V member 2, myosin regulatory light chain 9, and laminin receptor 1	Yao et al., 2008 [3]
			(iii) Decrease the ROS level	
NG-R1	Cultured human endothelial cells from different vascular sources		(iv) Increase the synthesis of t-PA and decrease PAI-1 activity	Zhang et al., 1997 [12]

PNS	H9c2 cells	Protecting myocardium cells from apoptosis	(i) Activate PI3K/Akt signaling pathway	Chen et al., 2011 [13]
PNS	H9c2 cells		(ii) Lower the levels of serum LDH, CK, and CK-MB and normalize myocardial superoxide dismutase, glutathione peroxidase, and catalase activities	Shi et al., 2007 [24]
PNS	Cultured cardiomyocytes		(iii) Alleviate intracellular calcium overload	Chen et al., 2005 [23]

Radix notoginseng formula	HUVEs	Promoting cardiac angiogenesis	Promote HUVEC proliferation and secretion of VEGF, expression of VEGFR-2 protein	Lei et al., 2010 [34]

Ginsenoside Rg1	Rat cardiomyocyte H/R model	Antimyocardial ischemia and hypoxia effect	Reduce lactate dehydrogenase release and increased cell viability and reduce intracellular ROS and suppressed the intracellular [Ca(2+)] level	Zhu et al., 2009 [38]

Notoginseng extracts (ginsenoside Rg1 and ginsenoside Rb1)	DCs 2.4 cells	Inhibitory effect on the inflammatory responses	Inhibition of TNF-alpha production and inhibit LPS-stimulated cytokine production	Rhule et al., 2008 [46]

PNS	VSMCs	Inhibition of intimal hyperplasia and smooth muscle cell proliferation	(i) Inhibit the activation of PDGF-induced P-ERK1/2 and increase the content of MKP-1	Zhang et al., 2012 [49]
PNS	VSMCs		(ii) Upregulate p53, Bax, and caspase-3 expressions and downregulate Bcl-2 expression	Xu et al., 2011 [50]
PNS	VSMCs		(iii) Inhibit the VSMC proliferation induced by hyperlipidemia serum	Lin et al., 1993 [51]
PNS	VSMCs		(iv) Inhibit the VSMC proliferation induced by hyperlipidemia serum	Wang et al., 2006 [8]

PNS	ApoE-KO mice	Antiatherosclerosis effect	(i) Lower the serum levels of lipid and oxLDL, ratio of plaque area to vessel area, and expression of CD40 and MMP-9	Liu et al., 2009 [53]
PNS	HUVEC		(ii) Improve its activity, elevate the adhesion rate with monocytes, and increase the protein expression of ICAM-1	Qin et al., 2008 [54]
			(iii) Decrease the mRNA expression levels of monocyte chemoattractant protein-1 and nuclear factor-kappaB/p65	Liu et al., 2010 [55]
PNS, ginsenoside Rg1, and ginsenoside Rb1	HCAECs		(iv) Inhibit TNF-alpha-induced NF-kappaB activation	Wang et al., 2011 [59]
PNS	HUVECs		(v) Regulate the VEGF-KDR/Flk-1 and PI3K-Akt-eNOS signaling pathways	Hong et al., 2009 [60]

PNS	Endothelial cells	Vasodilative effect	(i) Increase of Ca2+ level via the receptor-operated Ca2+ channels in the presence of ACh or the nonselective cation channels opened by CPA	C. Y. Kwan, and T. K. Kwan, 2000 [65]
Ginsenoside-Rd	VSMCs		(ii) Inhibit Ca2+ entry through ROCC and SOCC without effects on VDCC and Ca2+ release	Guan et al., 2006 [66]
Ginsenoside-Rd	BASMCs		(iii) Inhibit cell proliferation and reverse basilar artery remodeling	Li et al., 2012 [67]
Rb1 and Rg1	Endothelial cell		(iv) Increase endothelial-dependent vessel dilatation through the activation of NO by modulating the PI3K/Akt/eNOS pathway and l-arginine transport	Pan et al., 2012 [68]

PNS	Cultured myocardial cells	Inhibition of left ventricular remodeling	Inhibitory action on neurohormonal factor NE	Zhou et al., 2005 [71]

NG: notoginsengnosides; NG-R1: notoginsenoside R1; H/R: hypoxia/reoxygenation; DCs: dendritic cells; VSMCs: vascular smooth muscle cells; apoE-KO: apolipoprotein E-knockout; HUVEC: human umbilical vascular endothelial cells; HCAECs: human coronary artery endothelial cells; BASMCs: basilar artery smooth muscle cells; PRP: platelet rich plasma; Grb2: growth factor receptor-bound protein 2; ROS: reactive oxygen species; t-PA: plasminogen activator; PAI-1: plasminogen activator inhibitor-1; oxLDL: oxidized low density lipoprotein; ICAM-1: intercellular adhesion molecule-1; Ach: acetylcholine; CPA: cyclopiazonic acid; ROCC: receptor-operated; SOCC: store-operated; VDCC: voltage-dependent inward Ca2+ current.

**Table 2:** Cardiovascular effects of PNS (Rb1, Rg1, and R1) in vivo research.

Compounds	Organ/animals	Effects		Study
Raw and steamed extracts	Rats blood	Antiplatelet, anticoagulant, and antithrombotic effect protecting myocardium cells from apoptosis	(i) Prolong rat bleeding time	Lau et al., 2009 [11]
PNS	Rats heart		(ii) Activate PI3K/Akt signaling pathway and improve the left ventricular EF, left ventricular FS, LVDd, and LVDs at end systole	Chen et al., 2011 [13]
PNS (Rb1, Rg1)	Guinea pig heart		(iii) Inhibit the total myocardial ATPase	Chen et al., 1994 [25]

PNS	SD rats	Antimyocardial ischemia and hypoxia effect	Decrease MDA and elevated SOD activity and decrease cytokines such as TNF-*α*, CRP, and IL-1*β*	Han et al., 2013 [37]

Radix Notoginseng	Sprague-Dawley rats	Lipid-lowering effect	Decline in serum levels of TC, triglycerides, and low-density lipoprotein-cholesterol, with an increase in serum high-density lipoprotein-cholesterol levels; reduced levels of hepatic HMG-CoA reductase	Xia et al., 2011 [45]

Panax notoginseng root	Sprague-Dawley rats	Inhibition of Intimal Hyperplasia and smooth muscle cell proliferation	(i) Inhibit the vascular intimal hyperplasia and lower the expression of PCNA, cyclin E, cyclinD1, FN, and MMP-9	Wu et al., 2010 [47]
PNS	Rats heart		(ii) Lower the IA, IT, HRIA, the ratio of intimal/mesolamella area and thickness, and the expression of PCNA	Wang et al., 2009 [48]

PNS	Rabbits	Antiatherosclerosis effect	(i) Decrease the mRNA expression levels of monocyte chemoattractant protein-1 and nuclear factor-kappaB/p65 in the aorta wall after 8 weeks of treatment in rabbits and regulate the blood lipid profile	Liu et al., 2010 [55]
PNS	Rats heart		(ii) Enhance transcriptional activation of the LXRalpha gene promoter, subsequently upregulated of ABCA1 and ABCG1, and inhibit NF-kappaB DNA binding activity	Fan et al., 2012 [56]
PNS	Rats		(iii) Reduce the gene expression of some inflammatory factors, such as integrins, IL-18, IL-1beta, and matrix MMP-2 and MMP-9, increase the expression of IkappaBalpha, and attenuate the expression of NF-kappaB/p65	Zhang et al., 2008 [57]
PNS	Rats		(iv) Suppress phosphorylation of FAK on threonine 397, integrins expression, and NF-kappaB translocation	Yuan et al., 2011 [58]
PNS	Zebrafish		(v) Promote changes in the subintestinal vessels	Hong et al., 2009 [60]

Rg1	Open-chest dogs	Antiarrhythmia effect	Prolong SNRT, AVWCL, and VERP and increase VFT	Wu et al., 1995 [63]

PNS	Rats	Inhibition of left ventricular remodeling	(i) Inhibit the markers of cardiac hypertrophy including HW/BW, LVW/BW, and the MD	Zhou et al., 2005 [71]
PNS	SHR		(ii) Increase the effect of calcium pump on the membrane of sarcoplasmic reticulum, decrease the myocardial intracellular Ca2+, and reduce the mass of the left ventricular muscle	Feng and Jiang, 1998 [72]
PNS	Rats		(iii) Stimulate ACE2 to inhibit the expression of TNF-alpha and enhance the antioxidance	Guo et al., 2010 [73]

EF: ejection fractions; FS: fractional shortening; LVDd: left ventricular dimensions at end diastole; LVDs: left ventricular dimensions; MDA: malondialdehyde; SOD: superoxide dismutase; TNF-*α*: tumor necrosis factor-*α*; CRP: C-reactive protein; IL-1*β*: interleukin-1*β*; TC: total cholesterol; IA: intimal area; IT: intimal thickness; HRIA: hyperplasia ratio of intimal area; PCNA: proliferating cell nuclear antigen; ABCA1: ATP-binding cassette A1; ABCG1: ATP-binding cassette G1; IL: interleukin; MMP-2: metalloproteinases-2; SNRT: sinus node recovery time; AVWCL: AV conduction Wenckebach cycle length; VERP: ventricular effective refractory period; VFT: increase ventricular fibrillation threshold; HW/BW: weight/body weight; LVW/BW: left ventricular weight/body weight; MD: myofibril diameters.
